# Serological evidence for human exposure to *Bacillus cereus* biovar *anthracis* in the villages around Taï National Park, Côte d’Ivoire

**DOI:** 10.1371/journal.pntd.0008292

**Published:** 2020-05-14

**Authors:** Susann Dupke, Grit Schubert, Félicité Beudjé, Anne Barduhn, Maude Pauly, Emmanuel Couacy-Hymann, Roland Grunow, Chantal Akoua-Koffi, Fabian H. Leendertz, Silke R. Klee

**Affiliations:** 1 Robert Koch Institute, Centre for Biological Threats and Special Pathogens, ZBS 2: Highly Pathogenic Microorganisms, Berlin, Germany; 2 Robert Koch Institute, P3: Epidemiology of Highly Pathogenic Microorganisms, Berlin, Germany; 3 Laboratoire National d’Appui au Développement Agricole/Laboratoire central de Pathologie Animale, Bingerville, Côte d’Ivoire; 4 Centre de Recherche pour le Développement, Université Alassane Ouattara, Bouaké, Côte d’Ivoire; Stanford University, UNITED STATES

## Abstract

*Bacillus cereus* biovar *anthracis* (*Bcbva*) is an untypical anthrax-causing pathogen responsible for high wildlife mortality in Taï National Park (TNP), Côte d’Ivoire. However, nothing is known about its effect on the rural population living in the region bordering TNP. Contact to bushmeat is a known risk factor for exposure to a variety of zoonotic pathogens, but no human infections with *Bcbva* were noted so far. Therefore, we performed a retrospective seroprevalence analysis with sera from 1,386 study volunteers. We used assays which detect antibodies against the protective antigen PA, which is synthesized by both *Bcbva* and classic *B*. *anthracis*, and against the recently described antigen pXO2-60, a 35-kDa protein only produced by *Bcbva*. We found a high seroprevalence (22.37%) of antibodies against PA, and approximately half of those sera (10.46%) were also positive for the *Bcbva*-specific antigen pXO2-60. All sera negative for PA were also negative for antibodies against pXO2-60, confirming specificity and suitability of the PA/pXO2-60 combined serological assay. The fact that a large fraction of sera was positive for PA but negative for pXO2-60 can most likely be explained by lower immunogenicity of pXO2-60, but exposure to classic *B*. *anthracis* cannot be excluded. As only *Bcbva* has been detected in the TNP area so far, exposure to *Bcbva* can be suspected from the presence of antibodies against PA alone. In a questionnaire, most study participants reported contact to bushmeat and livestock carcasses. Unfortunately, risk factor analysis indicated that neither animal contacts, sex, age, nor country of origin were significant predictors of *Bcbva* seroprevalence. Nevertheless, our study added to an assessment of the distribution of *Bcbva* and its impact on the human population, and our data can serve to raise awareness of anthrax in the affected regions.

## Author summary

Anthrax is a zoonotic disease transmitted from animals to humans and normally caused by *B*. *anthracis* mainly in savanna regions. However, untypical bacteria named *Bacillus cereus* biovar *anthracis* (*Bcbva*) were detected in a variety of wild animals in the rain forest region of the Taï National Park (TNP) in Côte d’Ivoire. No anthrax infections in humans living in the region around TNP were reported until now. Therefore, we assessed exposure to the pathogen by analysis of sera from human volunteers for the presence of antibodies against the protective antigen (PA), which is produced by *B*. *anthracis* and *Bcbva*, and against the *Bcbva*-specific protein pXO2-60. We found antibodies against PA in more than 20% of sera from humans living in the TNP region, and around 10% possessed also antibodies against pXO2-60, confirming exposure to *Bcbva*. As only *Bcbva*, but not classic *B*. *anthracis* was found in TNP, we assume that the majority of humans had contact with *Bcbva* and that pXO2-60 is less immunogenic than PA. Although most people reported animal contacts, there was no statistically significant correlation with the presence of antibodies against *Bcbva*. Nevertheless, our study confirmed that *Bcbva* represents a danger for humans living in the affected area.

## Introduction

While the zoonotic potential and global importance of classic *B*. *anthracis* for human health is widely recognized [1, 2], little is known on the epidemiology of the “rainforest” anthrax-like disease caused by *Bacillus cereus* biovar *anthracis* (*Bcbva*). *Bcbva* has been shown to cause disease in a large number of wild animals belonging to different species, including chimpanzees, gorillas, elephants, different monkeys and duikers, in rainforest regions of Africa [3–5]. In fact, 40% of all wildlife carcasses found in Taï National Park (TNP), Côte d’Ivoire (CIV), tested positive for *Bcbva* with molecular or microbiological detection methods [4]. In contrast, to date no carcasses have been found to be positive for classic *B*. *anthracis* in TNP.

Most commonly humans contract classic anthrax from exposure through skin contact when handling infected animals or carcasses, their skins, wool, etc. (cutaneous anthrax), by ingestion of contaminated meat (gastrointestinal anthrax) or by inhalation of spore contaminated dust (pulmonary anthrax), e.g. when working with goat hair or wool [6–8]. Handling and consumption of bushmeat, either found dead in the forest or hunted, has previously been shown to play an important role for transmission of zoonotic pathogens to humans in the region near TNP and elsewhere [9–11]. While it is conceivable that bushmeat from animals infected with *Bcbva* is a source of infection with this pathogen for the local human population at Taï, to date there is no evidence for the occurrence of anthrax-like diseases caused by *Bcbva* among humans inhabiting this region.

Serological screening allows retrospective identification of areas affected by zoonotic diseases like anthrax, because the presence of antibodies can point to previous infection or exposure. This plays an important role especially in resource-limited countries. Here, outbreaks are only rarely officially reported and isolated anthrax cases in humans and livestock are hardly ever reported [12]. Detailed studies on the immune response after symptomatic or asymptomatic infection with *B*. *anthracis* revealed that antibody production against the anthrax toxin components protective antigen (PA) and lethal factor (LF) was dominant compared to edema factor (EF) [13–20]. Anti-PA antibodies had toxin-neutralizing and protective capacities; therefore PA is the main component of vaccines against anthrax [21, 22]. Furthermore, LF contributes to the immunization process, which could be shown in vaccinated persons, but most importantly in cutaneous anthrax patients [18, 23].

We have shown that *Bcbva* appeared as bacterium containing a *B*. *cereus-*like chromosomal background and both virulence plasmids pXO1 and pXO2 of *B*. *anthracis*. Therefore, *Bcbva* is able to produce a capsule and the three toxin components PA, LF, and EF [24, 25]. Furthermore, similar virulence of *Bcbva* and classic *B*. *anthracis* was shown in small animal models [26] and was supported by the high lethality of the disease for infected wildlife in TNP [4, 27]. Vaccination of mice with a vaccine formula consisting of formaldehyde-inactivated *B*. *anthracis* spores and recombinant PA was shown to be protective against both *B*. *anthracis* and *Bcbva* [26]. Therefore, it is likely that antibodies against PA and LF are also produced in humans after infection with *Bcbva*, like those observed in mice after immunization with toxin-containing culture supernatant of *Bcbva* [28]. However, serological analyses based on antibodies against PA or LF cannot distinguish between an immune response against *B*. *anthracis* or *Bcbva*. Therefore, we recently described a pXO2-encoded protein specific for *Bcbva*, named pXO2-60 [28]. Based on *in silico* structural analysis, pXO2-60 might belong to the aerolysin family of pore-forming toxins [29], but so far the function of the protein is unclear. In *B*. *anthracis*, secretion is hindered due to a nonsense mutation in the sequence for the signal peptide that needs to be cleaved from the protein precursor. The specificity of pXO2-60 was confirmed by the lack of antibodies in human control sera from patients infected with or exposed to classic *B*. *anthracis*. However, the immunogenicity of the antigen in mice seems to be lower than that of PA [28].

In the present study we tested whether pXO2-60 can be used to detect specifically exposure to *Bcbva* in a large-scale screening of human sera. We therefore performed a seroprevalence study of *Bcbva* in 1,386 human sera from inhabitants of the TNP region in CIV collected in the years 2006–2007 and 2011–2013, by testing for antibodies against PA, LF, and pXO2-60 through ELISA and Western Blot (WB) analysis. Potential demographic and behavioral risk factors for human exposure to anthrax were explored through questionnaire analysis. We show that the serological testing scheme employed here is suitable for routine retrospective analysis of exposure to anthrax-causing bacteria.

## Material and methods

### Ethics statement

The research was conducted in the framework of two studies funded by the German Research Foundation (DFG), which focused on the transmission of zoonotic pathogens from wildlife and livestock to humans (LE1813/4-1) as well as on anthrax epidemiology and ecology (KL2521/1-1). The investigation was approved by the Ivorian ethics commission (CNER, permit no. 101 10/MSHP/CENR/P; 0077–3 MSLS/CNER). After thorough explanation of the study objective and procedures, all persons volunteering to participate signed an informed consent prior to being included. For cases who are minors, a parent or guardian provided written informed consent on their behalf. Results were communicated back to the population on a general level through two workshops, and the risk of bushmeat hunting and consumption of animals found dead was discussed.

### The study

**Fig 1:**
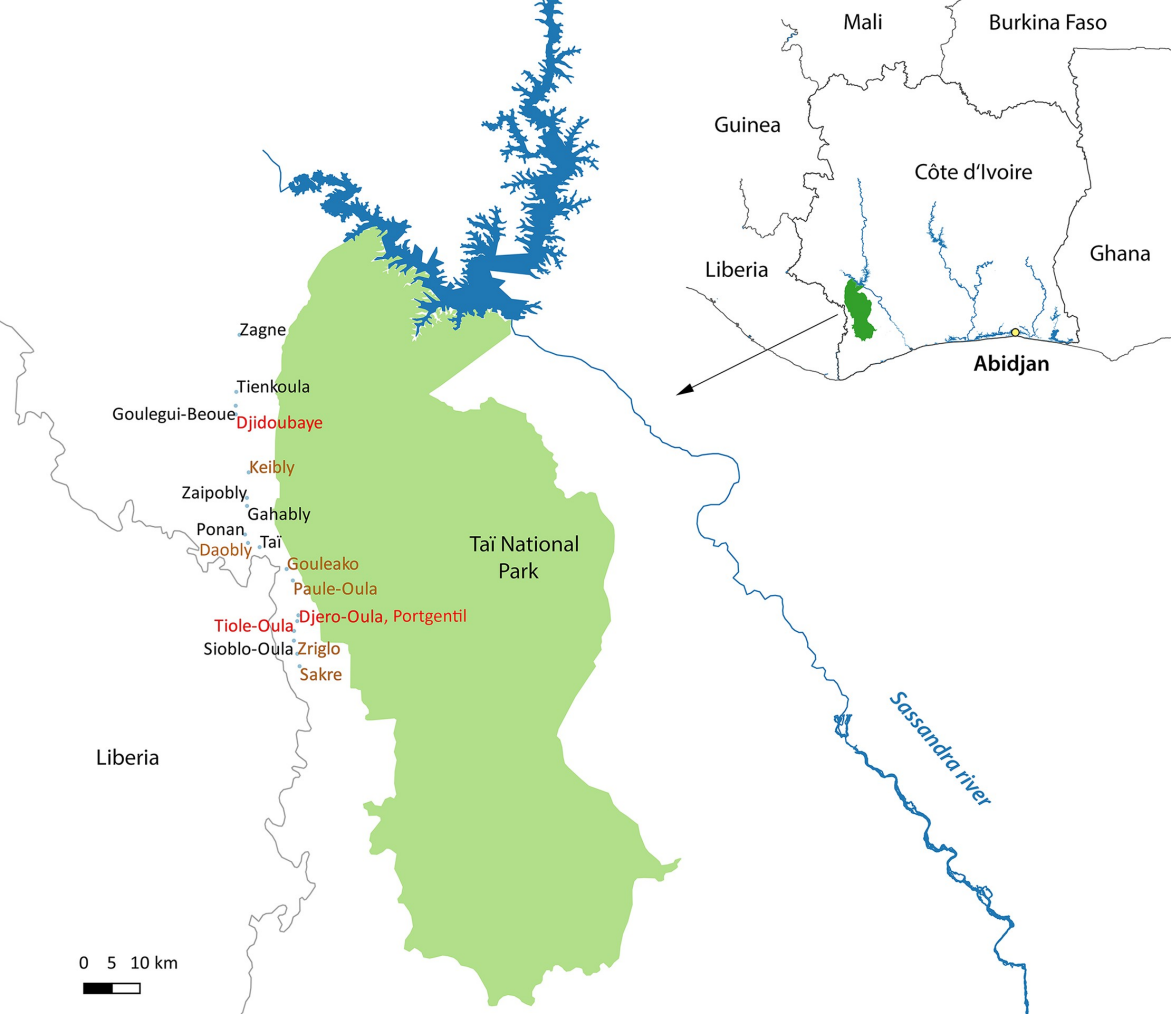
Study area at the border to Taï National Park. Taï National Park is located in the south-west of Côte d’Ivoire near the Liberian border. Participants from one town and 17 villages were included in the study. All sampling sites are located in similar proximity to the National Park, connected by a minor road. Villages were color coded by level of *Bcbva* seropositivity: black font, *Bcbva* seropositivity 0–10%; brown font, *Bcbva* seropositivity > 10%– 15%; red font, *Bcbva* seropositivity > 15%. The map has been created by the authors of the manuscript with the freely available software QGIS and edited with Adobe Photoshop. Shape files for Africa were freely available at http://maplibrary.org/library/index.htm.

During two sampling periods, 2006–2007 and 2011–2013, inhabitants from 17 villages (Daobly, Djero-Oula, Djiboubaye, Gahably, Gouléako, Goulégui-Béoué, Keibly, Paulé-Oula, Ponan, Portgentil, Sakré, Sioblo-Oula, Taï, Tiole-Oula, Tienkoula, Zaïpobly, and Ziriglo) and one town (Zagné) bordering TNP in Western Côte d’Ivoire were asked to participate in a study on zoonotic disease and associated risk factors for disease transmission. The number of collected samples per sites varied from 16 (Zagné) up to 152 (Keibly). All sampling locations are sited along the northern access road to TNP between the Liberian border and the park (Fig 1). In 2014, the number of inhabitants ranged from 1,000 up to 48,000 in Zagné, which is the largest town in the area [30]. The study objectives and methods were thoroughly explained to the local population by local health authorities and health workers. For each individual study participant, the objectives and methods were explained again prior to sampling. All participants signed informed consent forms, and data were treated anonymously in subsequent analyses.

### Collection of human serum samples

Blood from study participants was collected by venipuncture as described before including basic clinical examination [31]. Obtained sera were stored frozen until further analysis. Sera from persons vaccinated with the licensed anthrax vaccine BioThrax (Emergent Biosolutions, Gaithersburg, MD, USA) as well as a serum sample from a person with confirmed injectional anthrax infection [32] and from a worker in a wool factory exposed to *B*. *anthracis* [16] were used as positive controls for ELISA and WB analyses for detection of anti-PA and anti-LF antibodies as described recently [28]. Anonymous negative control sera were obtained from healthy German blood donors and from an occupational health service in accordance with German laws.

### Serological testing

#### ELISA for the detection of anti-PA and anti-pXO2-60 antibodies

PA- and pXO2-60-ELISA were performed as previously described with minor modifications [28]. Briefly, 0.1 μg of recombinant PA or 0.3 μg of recombinant pXO2-60 was coated per well. Blocking was performed with 4% skimmed milk powder (SMP) in phosphate buffered saline (PBS) containing 0.05% Tween-20 (PBS-T). Positive and negative control sera as well as test serum samples were diluted 1:1,000 in 1% SMP in PBS-T prior to addition to the plate. This comparatively high serum dilution was chosen after an evaluation step because of an elevated background OD value observed for African serum samples compared to European sera and to avoid false-positive results.

Goat anti-human antibody conjugated with horseradish peroxidase (HRPO, IgG+IgM+IgA (H+L), 0.8 mg/ml), diluted 1:10,000 was added, followed by colorimetrical detection of bound conjugate with 3,3’,5,5’-tetramethylbenzidine (TMB) at a wavelength of 450 nm with a 620 nm reference filter. All serum samples were tested in duplicate in two independent assays.

ELISA results below the arithmetic mean OD of 0.250 were considered as “negative”. Results above the mean OD of 0.350 were assumed to be “positive”, whereas all results between these two values were taken as “borderline”.

#### Western blot analysis

To confirm the results obtained in ELISA and to avoid false-positive results, we further examined the presence of antibodies against PA, LF, and pXO2-60 by using an in-house WB assay. Membrane stripes containing recombinant PA and pXO2-60 and in addition separate stripes with LF only were prepared as described previously and stored at 4°C prior to use [28]. To detect antibodies against PA, pXO2-60, and LF, membrane stripes were incubated with a 1:1,000 dilution (in 3% SMP in Tris-buffered saline (pH 7.6) with 0.1% Tween-20, TBS-T) of sera and subsequent binding of goat-anti-human HRPO-conjugate (1:10,000 in TBS-T). Antibody binding on stripes was visualized with precipitating TMB. Test sera were considered positive when they showed distinct signal bands for PA (83 kDa) and/or pXO2-60 (35 kDa).

In the same way, WB analysis was performed to detect anti-LF antibodies in test sera. Distinct signal bands of 89 kDa, corresponding to the size of the mature LF protein, were visible on membrane stripes when anti-LF antibodies were present in the serum sample.

### Survey of personal demographic information and contact to animals

We employed a standardized questionnaire with the rationale to examine potential risk factors for being exposed to *Bcbva*. We therefore gathered participants’ personal demographic information (sex, age, country of origin) as well as behavioral information on contact to wildlife and livestock as described by Mossoun and colleagues [9]. Non-human primates as well as wild ruminants (both consumed as bushmeat) have been described to carry *Bcbva* in the Taï region and are thus potential reservoirs for zoonotic transmission [4]. We therefore asked study participants whether they had been exposed to bushmeat during the hunting or killing, butchering, and cooking as proxy for such “risky” behaviors (S4 Table). We recorded those data separately for monkey, great ape, and wild ruminants, as those categories are readily recognized by the local human population. For domestic ruminants, only data on meat preparation (not killing/butchering) were available, separately for sheep, goat, and cattle. Within each sampling period (2006–2007 and 2011–2013), a team consisting of two trained researchers which was supported by two trained local interpreters conducted the interviews in French and if necessary also in commonly spoken dialects (e.g. Dioula, Guéré, or Oubi). In total, 1,386 participants were recruited for the study, with a sex ratio female: male of 1.29; and a median age of 31 years (min: 0, max: 95). 75.90% of participants were born in CIV, 20.18% in Burkina Faso, and 3.91% in other countries.

### Statistical analyses–Predictors of *Bcbva* seropositivity

To investigate what influenced the probability of being *Bcbva* seropositive, we used Generalized Linear Mixed Models (GLMM) [33] with binomial error structure and logit link function. Reactivity to pXO2-60 antigen was considered evidence of *Bcbva* exposure (“confirmed” cases), while reactivity only to PA was considered indicative of likely exposure to *Bcbva* (“suspected” cases). We therefore analyzed the data using two models with the following binary response variables: Model 1) reactive/not reactive to pXO2-60 antigen during WB analysis (confirmed cases), and Model 2) reactive/not reactive to PA antigen with or without pXO2-60 during WB analysis (confirmed and suspected cases combined). In both models, we included the covariate age and the fixed effects sex (female/male) and country of origin (Côte d’Ivoire, Burkina Faso, other) as well as contact to bushmeat (pooled for hunting, butchering and preparing monkey, chimpanzee, and duiker; “yes” if participants had been performing at least one of the three actions, otherwise “no”) and contact to domestic animals (pooled for preparing sheep, goat, or cattle; “yes” if participants had been engaged in meat preparation, otherwise “no”). Sampling village/town was included as random effect [34]. We attempted to account for random slopes of all fixed effects in the two models in order to keep type I error rate at the nominal level of 5% [34, 35]. Models were, however, unidentifiable and we subsequently removed the correlation parameters between random intercepts and random slopes terms and all random slopes except for contact to bushmeat. We excluded participants with missing values. Age was z-transformed to a mean of zero and a standard deviation of one.

All models were fitted as logistic models in R [36] using the function glmer of the R-package lme4 [37, 38]. Checks for model stability did not indicate influential subjects to exist. Variance Inflation Factors [39] did not indicate collinearity to be an issue.

The significance of the full model as compared to the null model comprising only the random effect was examined through a likelihood ratio test [implemented through the R-function anova with ‘argument test’ set to ‘Chisq’ [40, 41].

## Results

### Serological test validation and seroprevalence of *B*. *anthracis* and *Bcbva*

Sera of 1,386 study participants were pre-screened using a validated in-house ELISA for anti-PA antibodies, resulting in 496 sera showing a positive or borderline signal (mean, 95% CI). For confirmation, all 496 sera were tested in WB analysis using membrane stripes containing recombinant PA and recombinant pXO2-60. Out of the 496 serum samples, a total of 310 were confirmed as PA-positive by WB analysis (310/1386, 22.37%) and thus classified as “suspected” *Bcbva* exposure. 145 of these 496 samples additionally reacted with the pXO2-60 band and thus classified as “confirmed” *Bcbva* exposure (145/1386, 10.46%, S1 Fig). To confirm that other serum samples lacking anti-PA antibodies do not contain anti-pXO2-60 antibodies as well, we additionally analyzed a random subset of 48 sera from different villages tested negative for anti-PA antibodies in ELISA for pXO2-60 [28]. All these sera could be confirmed to lack antibodies against pXO2-60 when tested in ELISA and WB and antibodies against LF when tested in WB as well. Therefore, we can conclude that antibody reaction against pXO2-60 is specific for contact to *Bcbva* (which also raises anti-PA and/or anti-LF antibodies) and not the result of cross-reactivity after contact to other bacteria. Interestingly, we found six sera showing a positive or borderline signal for anti-PA antibodies in ELISA but being negative in WB confirmation for anti-PA. Nevertheless, a weak band was visible for anti-pXO2-60 antibodies on the same membrane stripe, confirmed by subsequent analysis in ELISA for this *Bcbva*-specific antigen. All six sera additionally contained antibodies against LF, which could be shown by WB analysis (S1 Fig). As negative control group (no exposition to anthrax expected) we tested 20 sera from German blood donors which showed no reaction against PA, LF, or pXO2-60 in ELISA and WB analysis. All five positive control sera tested did not contain antibodies against pXO2-60, but were clearly positive for anti-PA antibodies. ELISA raw data are shown in S3 Table as well as S2 Fig. As indicated in Table 1, seroprevalence varied broadly among the villages sampled, from 10.71% anti-PA and 3.57% anti-pXO2-60 prevalence in Taï up to 38.71% anti-PA and 19.35% anti-pXO2-60 prevalence in Djero-Oula, respectively.

**Table 1:** Results overview of serological analyses of reactivity to PA and pXO2-60 antigen among serum samples from humans inhabiting the Taï region in Western Côte d’Ivoire.

Village	Sample no.	% suspected *Bcbva* exposure (no. of reactive sera to PA antigen in Western Blot) *	% confirmed *Bcbva* exposure (no of reactive sera to pXO2-60 antigen in Western Blot)
Zagné	16	12.50 (2)	0.00 (0)
Tienkoula	40	22.50 (10)	7.50 (6)
Goulégui-Béoué	55	36.36 (20)	9.09 (5)
Djidoubaye	40	27.50 (11)	17.50 (7)
Keibly	152	15.79 (24)	11.84 (18)
Zaïpobly	99	13.13 (13)	9.09 (9)
Gahably	151	13.25 (20)	5.30 (8)
Daobly	131	30.53 (40)	13.74 (18)
Ponan	120	14.17 (17)	8.33 (10)
Taï	56	10.71 (6)	3.57 (2)
Gouléako	119	27.70 (33)	11.80 (14)
Paulé-Oula	106	24.53 (26)	11.32 (12)
Portgentil	47	27.66 (13)	17.02 (8)
Djero-Oula	31	38.71 (12)	19.35 (6)
Tiele-Oula	38	26.32 (10)	15.79 (6)
Sioblo-Oula	35	28.57 (10)	5.71 (2)
Ziriglo	75	30.67 (23)	10.67 (8)
Sakré	75	28,00 (21)	12.00 (9)

* Including six sera positive for anti-LF antibodies but negative for anti-PA antibodies

### Human contact to wildlife and lifestock and individual level risk factors

Previous contact to bushmeat (monkeys, great apes, or wild ruminants) through hunting, butchering, or preparation was reported by 91.77% of the female and 66.67% of the male participants. Rates of contact to ruminant meat (i.e. cattle, sheep, goat) during food preparation were 80.71% for women and 36.33% for men. Using multivariate analysis, we assessed factors potentially affecting the risk of human exposure to *Bcbva*. None of the models was statistically significant (Model 1 with response variable reactive/not reactive to pXO2-60 antigen: likelihood ratio test: *χ*^*2*^ = 9.172, DF = 6, P = 0.164, N = 1172; Model 2 with response variable reactive/not reactive to PA antigen with or without pXO2-60 antigen: likelihood ratio test: *χ*^*2*^ = 6.095, DF = 6, P = 0.4130, N = 1172). This indicates that participants’ sex, age, country of origin, and contact to bushmeat or livestock are not significant predictors of *Bcbva* seroprevalence of humans in the Taï region.

## Discussion

*Bcbva* is endemic and found in wildlife carcasses throughout the year in the TNP. Here we show that *Bcbva* also affects the rural human population living in the region bordering the TNP in South-Western CIV. Exposure to *Bcbva* was shown using an innovative approach combining the routinely used and commercially available anti-PA antibody detection method with recently described anti-pXO2-60 antibody detection. All serum samples tested giving a negative result in PA-ELISA did not contain anti-pXO2-60 antibodies, which confirms the applicability of the ELISA as a useful screening tool for both anthrax-causing bacteria species. When handling small sample sizes, however, membrane stripes coated with the two antigens provide a fast and easy test. These stripes can be prepared in every standard laboratory and transferred to small laboratories in remote areas where only few reagents and equipment are available.

Out of 1,386 human serum samples tested here, 22.37% were found to be positive for the presence of anti-PA antibodies, while only approximately half of those sera (10.45%) also contained antibodies against the *Bcbva*-specific antigen pXO2-60. Seroprevalence varied widely among the different sampling locations, ranging from 10.71% up to 38.71% for anti-PA and 0% up to 19.35% for anti-pXO2-60 antibodies (Table 1). The substantial number of individuals positive for PA, but negative for pXO2-60, was possibly due to a lower immunogenicity of pXO2-60 compared to PA. This was also seen in immunization experiments with toxin-containing *Bcbva* culture supernatant where anti-pXO2-60-antibodies were only detected in three out of five mice [28]. Six of the 1,386 sera failed to react with recombinant PA on the WB membrane stripes despite a positive or borderline signal (OD values ranging from 0.3 to 1.6) in ELISA testing before. These six sera nevertheless contained antibodies against pXO2-60 and LF when tested in WB analyses, indicating exposure to *Bcbva*. It cannot be excluded that LF was more immunogenic for these individuals than PA, as was reported for patients infected with cutaneous anthrax [18]. Differences in anti-PA and -pXO2-60 prevalence might also be caused by different persistence patterns of these antibodies in patients’ blood. Quinn and colleagues showed that after inhalational anthrax, anti-PA antibodies were detectable for eight to 16 months after the onset of symptoms of six patients examined [13]. In addition, exposure by the cutaneous route does not lead to production of anti-PA antibodies in all affected individuals [13, 42]. We do not yet have any data on the antibody dynamic on anti-pXO2-60 antibodies. However, in light of these studies, the 10.45% seropositivity with the pXO2-60 marker may be seen as the minimum estimate (confirmed *Bcbva* exposure) while positivity of 22.37% with the PA marker represents a respective maximum estimate for *Bcbva* seroprevalence (confirmed and suspected cases). It should also be mentioned that the assay was validated for maximal specificity required for epidemiological studies rather than a high sensitivity which is most important for a diagnostic assay. As no true positive control sera from confirmed patients with anthrax infection caused by *Bcbva* were available, the cut-off of the screening ELISA was set using the mean value plus two SD of all sera (probable negative and probable positive), which additionally decreased the detection sensitivity, but increased the specificity. Thus, the obtained rate of seropositivity is most likely a conservative estimate.

A positive anti-PA antibody titer cannot distinguish between exposition to *Bcbva* or classic *B*. *anthracis*, but as the latter has not yet been detected in the rainforest region of TNP, we assume that the positive anti-PA titer is based on an immune response against *Bcbva* rather than *B*. *anthracis*. However, contact with *B*. *anthracis* cannot be completely ruled out, as is described below.

Study on the impact of anthrax on animal and human health in West African countries is scarce to date. Respective research focuses almost exclusively on countries with mass die-offs of wild ruminants caused by anthrax in large national parks like those in Namibia and South Africa [43–46], Tanzania [47, 48], but also Kenya [49] and Botswana [50]. Because of lacking systematic surveillance, only few data are available on the prevalence of anthrax in CIV, and only one putative isolate of classic *B*. *anthracis* has so far been described [51]. In 2013, five fatal human cases were reported from an outbreak in the north-east [52] near the border to Ghana, a country where classic *B*. *anthraci*s is present [53–55]. Beside the rare official reporting, regular outbreaks of anthrax most likely caused by classic *B*. *anthracis* occur in northern CIV every year after the rainy season in humans and livestock (E. Couacy-Hymann, pers. communication), while in the area of TNP only *Bcbva* was found so far [4, 27]. However, as far as we know the endemic regions of classic anthrax and *Bcbva* do not overlap geographically which might be due to the different climatic and environmental conditions given within a tropical rainforest and in savanna regions. It cannot be ruled out that a certain number of PA-positive/pXO2-60-negative individuals had been exposed in the suspected classic anthrax region comprising a savanna landscape and then migrated to the area surrounding TNP. As both inland and border-crossing migration is common in CIV, this must be always considered to be a likely possibility [56]. It is also possible that study participants may have had contact with classic *B*. *anthracis* before exposure to *Bcbva*. Here, the further exposition to the PA-producing bacterium may lead to enhanced antibody production resulting in a stronger PA-answer, while a single contact to *Bcbva* might not be sufficient for an immune response against pXO2-60. Also vaccination studies have shown earlier that antibody response to PA was positively affected by the number of doses [20, 57].

In TNP, *Bcbva* has been circulating among wildlife for decades [4]. In our study, most participants reported having had contact to bushmeat and livestock carcasses, providing ample opportunity for transmission of the bacteria. However, the study could not determine whether individuals had contact with diseased wild or domestic animals and even less whether animals showed anthrax-like symptoms or were infected with *Bcbva*. In addition, such contact varies greatly among sex and age [9], hinting at potential risk groups for exposure. 20% of participants had been born in Burkina Faso, where primarily dry savanna-type habitat fosters the occurrence of anthrax, outbreaks are frequently reported [58–62], and thus anthrax seroprevalence in humans is likely to be high. However, neither contact to bushmeat nor livestock, country of birth, sex, or age were predictors of detection rates of anti-PA or anti-pXO2-60 antibodies in humans inhabiting the region of TNP in Western CIV.

Multiple factors may obscure the signal of *Bcbva* exposure and could potentially hinder the identification of respective risk factors: 1) Exposition to *Bcbva* without production of antibodies against pXO2-60 is possible, as was seen for mice immunized with *Bcbva* culture supernatant. This highlights also the need for use of additional *Bcbva*-specific antigens in the serological assays. 2) Antibodies present against pXO2-60 confirm exposure to *Bcbva* but do not rule out previous exposure to classic *B*. *anthracis*. Again, a serological assay using a *B*. *anthracis*-specific antigen would be desirable for this purpose. 3) After cutaneous anthrax infection (which is most probable for exposed but untreated persons), antibody response against PA is not at 100% and not lasting long enough to permit prolonged detection [13]. In an investigation of 14 outbreaks in Central Bangladesh, Chakraborty and colleagues showed that only 34 of 45 persons had a detectable anti-PA titer after developing suspected cutaneous anthrax after having slaughtered and handled sick animals [42]. Thus, repeated exposure to classic *B*. *anthracis* or *Bcbva* might be necessary to detect respective antibodies long-term, and true exposure to anthrax might be higher than that shown in our study. In this light, the rates of detection of anti-PA and anti-pXO2-60 antibodies found here are very conservative, potentially short-term indicators of previous anthrax exposure.

In sum, our results show that the PA/pXO2-60 WB analysis is a suitable tool to reliably detect antibodies against *Bcbva* in humans, indicating an infection with these anthrax-causing bacteria. Identifying factors that may facilitate an exposure to (infectious?) anthrax is however challenging, as more research on the long-term immunological footprint in anthrax-infected individuals is needed.

## Supporting information

S1 Fig Examples of antibody reactions of human sera with *Bcbva* antigens on membrane stripes. Stripes 1–3: three different sera which are both PA- and pXO2-60-positive; stripe 4: PA-positive serum; stripe 5: PA- and pXO2-60-negative serum; stripes 6 and 7: same serum which is PA-negative and pXO2-60-positive (6) and LF-positive (7). Signal band sizes: PA 83 kDa, pXO2-60 35 kDa, LF 89 kDa.
(TIF)

S2 Fig Distribution of mean PA-ELISA OD values per sample. The figure is showing the respective 25th– 75th percentile (boxes) with the line indicating the median, as well as minimum and maximum ranges (whiskers). Test specimens with OD < 0.25 (N = 890) were classified as PA-negative, test specimens with OD > 0.35 (N = 303) were classified as PA-positive. Specimens with “borderline” OD values (0.25–0.35, N = 193) are not included in this figure. Subsequent confirmation by Western Blot analysis is not taken into account here.
(TIF)

S1 Table Proportions of sera reactive to PA and pXO2-60 antigen analyzed by sex, country of birth, contact to bushmeat, and contact to livestock. (DOCX)

S2 Table STROBE checklist. (DOC)

S3 Table Results of ELISA testing and Western Blot analyses performed on anti-PA, anti-pXO2-60 and anti-LF antibodies. Mean OD values were calculated from duplicates of two independent anti-PA ELISA. All serum samples giving a mean OD value higher than or equal to 0.250 were tested in a confirmatory Western Blot analysis for anti-PA as well as anti-pXO2-60 antibodies. Anti-LF antibody testing was performed randomly.
(XLSX)

S4 Table Standardized questions on bushmeat consumption used in interviews with study participants in Western Côte d’Ivoire. Questions were posed in French or local dialect, and are translated into standard English for the purpose of this table. We asked study participants for contact to bushmeat animal groups which are readily distinguished by the local population. Those are monkeys (French word used: singe), chimpanzees, and wild ruminants (French words used: biche or antilope). Contact to bushmeat was categorized as hunting, dismembering and cooking. We initially divided those categories further by asking if contact was happening daily / weekly / monthly / on special occasion in order to examine the individual frequency of bushmeat contact. However, answers on such time-dependent events tended to be unrealistic (e.g., cooking the very rare and highly endangered genus Chimpanzee daily). We therefore rated answers with “yes” if any of those categories was answered with „yes“, and “no” if all answers were negative. We applied the same procedures for contact to the domestic animal groups sheep, goat, and cattle, however for those restricted the questions to meat preparation.
(DOCX)
