# Supplementary figures and images for: Serological evidence for human exposure to Bacillus cereus biovar anthracis in the villages around Taï National Park, Côte d’Ivoire
Source: PLoS Negl Trop Dis. 2020 May 14;14(5):e0008292. doi: 10.1371/journal.pntd.0008292 (PMC7224451; doi:10.1371/journal.pntd.0008292)

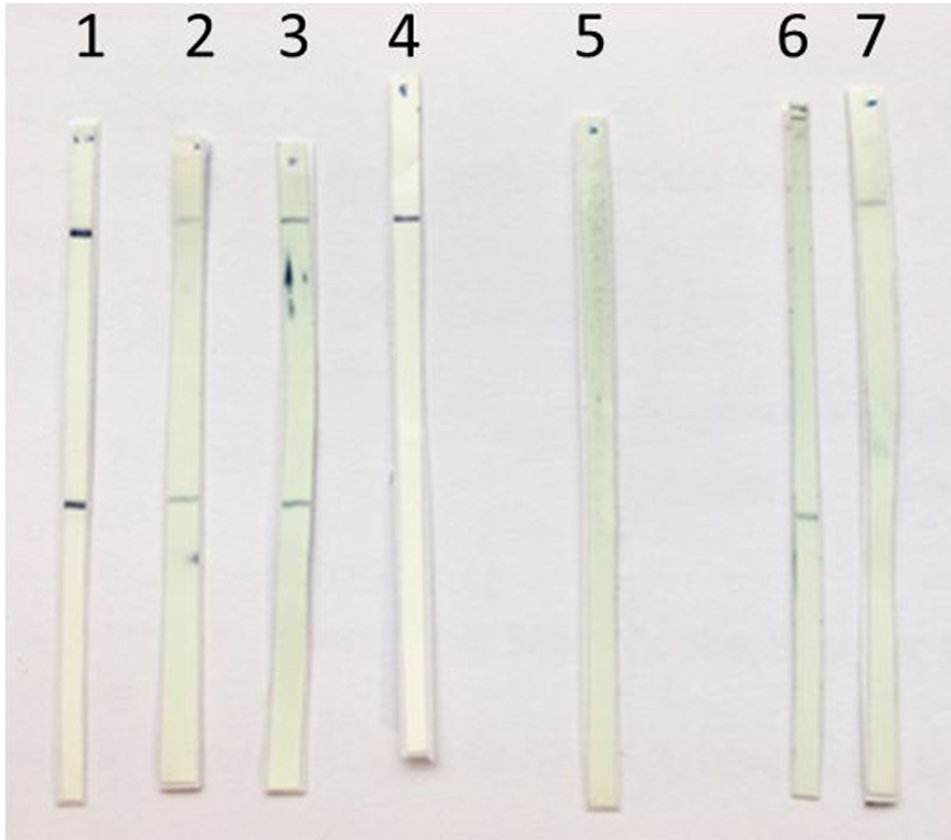

Supplement: S1 Fig — Stripes 1–3: three different sera which are both PA- and pXO2-60-positive; stripe 4: PA-positive serum; stripe 5: PA- and pXO2-60-negative serum; stripes 6 and 7: same serum which is PA-negative and pXO2-60-positive (6) and LF-positive (7). Signal band sizes: PA 83 kDa, pXO2-60 35 kDa, LF 89 kDa. (TIF) [file pntd.0008292.s001.tif]

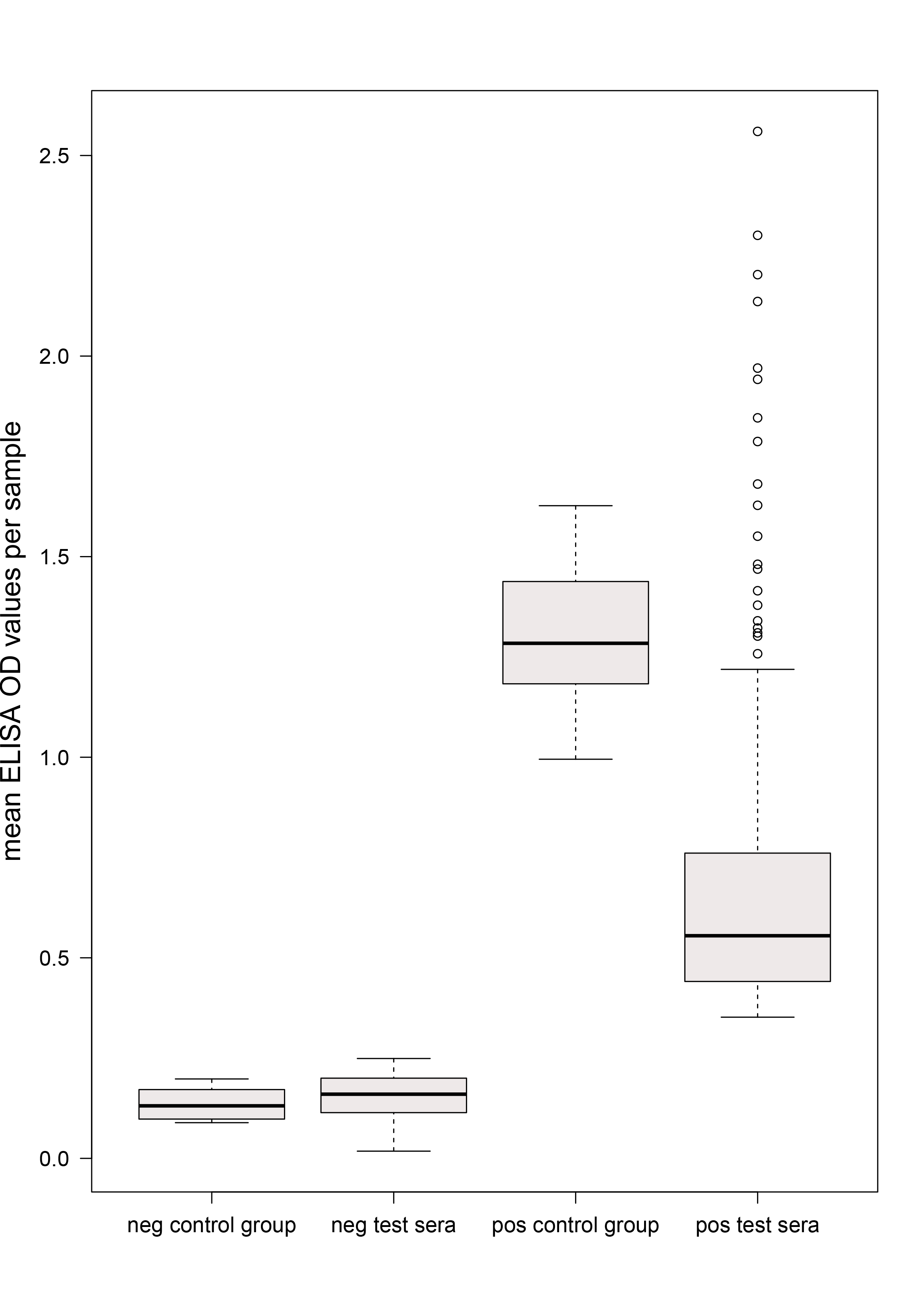

Supplement: S2 Fig — The figure is showing the respective 25th– 75th percentile (boxes) with the line indicating the median, as well as minimum and maximum ranges (whiskers). Test specimens with OD < 0.25 (N = 890) were classified as PA-negative, test specimens with OD > 0.35 (N = 303) were classified as PA-positive. Specimens with “borderline” OD values (0.25–0.35, N = 193) are not included in this figure. Subsequent confirmation by Western Blot analysis is not taken into account here. (TIF) [file pntd.0008292.s002.tif]
