# Supplementary material for: Serological evidence for human exposure to Bacillus cereus biovar anthracis in the villages around Taï National Park, Côte d’Ivoire
Source: PLoS Negl Trop Dis. 2020 May 14;14(5):e0008292. doi: 10.1371/journal.pntd.0008292 (PMC7224451; doi:10.1371/journal.pntd.0008292)
Supplement: S1 Table — (DOCX) [file pntd.0008292.s003.docx]

**S1 Table.** Proportions of sera reactive to PA and pXO2-60 antigen analyzed by sex, country of birth, contact to bushmeat, and contact to livestock.

| Risk factor | % reactive to PA antigen in Western Blot (suspected exposure to *Bcbva*)* | % reactive to PA and pXO2-60 antigen in Western Blot (confirmed exposure to *Bcbva*) |
| --- | --- | --- |
| *Sex* |  |  |
| F | 23.6 | 9.7 |
| M | 22.1 | 11.4 |
| *Country of birth* |  |  |
| BF | 20.2 | 6.5 |
| CIV | 23.7 | 11.4 |
| other | 23.5 | 9.8 |
| *Bushmeat contact* |  |  |
| no | 20.1 | 10.7 |
| yes | 24.5 | 11.0 |
| *Livestock contact* |  |  |
| no | 24.0 | 11.6 |
| yes | 22.0 | 9.5 |

*includes sera reactive to PA with or without reactivity to pXO2-60
