# Supplementary material for: Serological evidence for human exposure to Bacillus cereus biovar anthracis in the villages around Taï National Park, Côte d’Ivoire
Source: PLoS Negl Trop Dis. 2020 May 14;14(5):e0008292. doi: 10.1371/journal.pntd.0008292 (PMC7224451; doi:10.1371/journal.pntd.0008292)
Supplement: S2 Table — (DOC) [file pntd.0008292.s004.doc]

**Dupke, Schubert, Beudjé, Barduhn, Pauly, Couacy-Hymann, Grunow, Akoua-Koffi, Leendertz and Klee:**

**Serological Evidence for human exposure to Bacillus cereus biovar anthracis in the villages around Taï National Park**

STROBE Statement—Checklist of items that should be included in reports of ***cross-sectional studies***

|  | Item No | Recommendation |
| --- | --- | --- |
| **Title and abstract** | 1 | (*a*) Indicate the study’s design with a commonly used term in the title or the abstract (abstract) |
|  |  | (*b*) Provide in the abstract an informative and balanced summary of what was done and what was found (abstract) |
| Introduction | | |
| Background/rationale | 2 | Explain the scientific background and rationale for the investigation being reported (introduction, paragraph 1 and 2) |
| Objectives | 3 | State specific objectives, including any prespecified hypotheses (introduction, last paragraph) |
| Methods | | |
| Study design | 4 | Present key elements of study design early in the paper (materials, sub-section 1 “The study”) |
| Setting | 5 | Describe the setting, locations, and relevant dates, including periods of recruitment, exposure, follow-up, and data collection (materials, sub-section 1 “The study”) |
| Participants | 6 | *(*a) Give the eligibility criteria, and the sources and methods of selection of participants:  Any inhabitant of the villages detailed in the materials, sub-section 1 “The study”, that volunteered to participate was included in the study. |
| Variables | 7 | Clearly define all outcomes, exposures, predictors, potential confounders, and effect modifiers. Give diagnostic criteria, if applicable  All outcomes and predictors used in statistical analyses are explained in the materials, sub-section 6 “Statistical Analyses - Predictors of Bcbva seropositivity”. The outcomes of laboratory analyses were not linked to clinical presentations of the study participants. |
| Data sources/ measurement | 8* | For each variable of interest, give sources of data and details of methods of assessment (measurement). Describe comparability of assessment methods if there is more than one group  All variables used in statistical analyses detailed in the materials, sub-section 5 “Survey of personal demographic information and contact to animals” were determined by use of a standardized questionnaire completed by all participants. |
| Bias | 9 | Describe any efforts to address potential sources of bias  We included any inhabitant of the villages detailed in the materials, sub-section 1 “The study”, that volunteered to participate into the study. We therefore could not influence the composition of the study group’s composition in this cross-sectional study. Basic demographic parameters are given in the materials, sub-section 5 “Survey of personal demographic information and contact to animals”. |
| Study size | 10 | Explain how the study size was arrived at  We included any inhabitant of the villages detailed in the materials,sub-section 1 “The study”, that volunteered to participate into the study. |
| Quantitative variables | 11 | Explain how quantitative variables were handled in the analyses. If applicable, describe which groupings were chosen and why  The quantitative variable “age” was included as covariate in the statistical analysis of the data. The number of participants per village/town was included as random effect into statistical models. Materials, sub-section 6 “Statistical Analyses - Predictors of Bcbva seropositivity” |
| Statistical methods | 12 | (*a*) Describe all statistical methods, including those used to control for confounding materials, sub-section 6 “Statistical Analyses - Predictors of Bcbva seropositivity” |
|  |  | (*b*) Describe any methods used to examine subgroups and interactions  materials, sub-section 6 “Statistical Analyses - Predictors of Bcbva seropositivity” |
|  |  | (*c*) Explain how missing data were addressed  We excluded participants with missing values from multivariate statistical analyses. (materials, sub-section 6 “Statistical Analyses - Predictors of Bcbva seropositivity”) |
|  |  | (*d*) If applicable, describe analytical methods taking account of sampling strategy  The number of participants per village/town was included as random effect into statistical models. (materials, sub-section 6 “Statistical Analyses - Predictors of Bcbva seropositivity”) |
|  |  | (*e*) Describe any sensitivity analyses  n.a. |
| Results | | |
| Participants | 13* | (a) Report numbers of individuals at each stage of study—eg numbers potentially eligible, examined for eligibility, confirmed eligible, included in the study, completing follow-up, and analysed  results, sub-section 1 “Serological test validation and seroprevalence of B. anthracis and Bcvba” and 2 “Human contact to wildlife and lifestock and individual level risk factors” |
|  |  | (b) Give reasons for non-participation at each stage  n.a. |
|  |  | (c) Consider use of a flow diagram  n.a. |
| Descriptive data | 14* | (a) Give characteristics of study participants (eg demographic, clinical, social) and information on exposures and potential confounders  results, sub-section 2 “Human contact to wildlife and lifestock and individual level risk factors” |
|  |  | (b) Indicate number of participants with missing data for each variable of interest  The sample size used, and thus the number patients excluded with missing values upon multivariate analyses, is given in the results, sub-section 2 “Human contact to wildlife and lifestock and individual level risk factors” |
| Outcome data | 15* | Report numbers of outcome events or summary measures  results, sub-section 1, table 1 |
| Main results | 16 | *(*a) Give unadjusted estimates and, if applicable, confounder-adjusted estimates and their precision (eg, 95% confidence interval). Make clear which confounders were adjusted for and why they were included  n.a. |
|  |  | (*b*) Report category boundaries when continuous variables were categorized  n.a. |
|  |  | (*c*) If relevant, consider translating estimates of relative risk into absolute risk for a meaningful time period  n.a. |
| Other analyses | 17 | Report other analyses done—eg analyses of subgroups and interactions, and sensitivity analyses |
| Discussion | | |
| Key results | 18 | Summarise key results with reference to study objectives  discussion, paragraph 2 |
| Limitations | 19 | Discuss limitations of the study, taking into account sources of potential bias or imprecision. Discuss both direction and magnitude of any potential bias  discussions, paragraphs 2, 3, 4 |
| Interpretation | 20 | Give a cautious overall interpretation of results considering objectives, limitations, multiplicity of analyses, results from similar studies, and other relevant evidence discussion, paragraph 4 |
| Generalisability | 21 | Discuss the generalisability (external validity) of the study results  discussion, paragraph 4 and 5 |
| Other information | | |
| Funding | 22 | Give the source of funding and the role of the funders for the present study and, if applicable, for the original study on which the present article is based  not included in the manuscript, information given at “additional information” during submission process |

*Give information separately for exposed and unexposed groups.

**Note:** An Explanation and Elaboration article discusses each checklist item and gives methodological background and published examples of transparent reporting. The STROBE checklist is best used in conjunction with this article (freely available on the Web sites of PLoS Medicine at http://www.plosmedicine.org/, Annals of Internal Medicine at http://www.annals.org/, and Epidemiology at http://www.epidem.com/). Information on the STROBE Initiative is available at www.strobe-statement.org.
