# Supplementary material for: Serological evidence for human exposure to Bacillus cereus biovar anthracis in the villages around Taï National Park, Côte d’Ivoire
Source: PLoS Negl Trop Dis. 2020 May 14;14(5):e0008292. doi: 10.1371/journal.pntd.0008292 (PMC7224451; doi:10.1371/journal.pntd.0008292)
Supplement: S4 Table — Questions were posed in French or local dialect, and are translated into standard English for the purpose of this table. We asked study participants for contact to bushmeat animal groups which are readily distinguished by the local population. Those are monkeys (French word used: singe), chimpanzees, and wild ruminants (French words used: biche or antilope). Contact to bushmeat was categorized as hunting, dismembering and cooking. We initially divided those categories further by asking if contact was happening daily / weekly / monthly / on special occasion in order to examine the individual frequency of bushmeat contact. However, answers on such time-dependent events tended to be unrealistic (e.g., cooking the very rare and highly endangered genus Chimpanzee daily). We therefore rated answers with “yes” if any of those categories was answered with „yes“, and “no” if all answers were negative. We applied the same procedures for contact to the domestic animal groups sheep, goat, and cattle, however for those restricted the questions to meat preparation. (DOCX) [file pntd.0008292.s006.docx]

| **Number** | **Question** |
| --- | --- |
| 1 | Are you hunting monkeys daily / weekly / monthly / on special occasion? |
| 2 | Are you butchering monkeys daily / weekly / monthly / on special occasion? |
| 3 | Are you preparing monkeys daily / weekly / monthly / on special occasion? |
| 4 | Are you hunting chimpanzees daily / weekly / monthly / on special occasion? |
| 5 | Are you butchering chimpanzees daily / weekly / monthly / on special occasion? |
| 6 | Are you preparing chimpanzees daily / weekly / monthly / on special occasion? |
| 7 | Are you hunting wild ruminants daily / weekly / monthly / on special occasion? |
| 8 | Are you butchering wild ruminants daily / weekly / monthly / on special occasion? |
| 9 | Are you preparing wild ruminants daily / weekly / monthly / on special occasion? |
| 10 | Are you preparing sheep daily / weekly / monthly / on special occasion? |
| 11 | Are you preparing goat daily / weekly / monthly / on special occasion? |
| 12 | Are you preparing cattle daily / weekly / monthly / on special occasion? |
